# Intraoperative Fracture during the Insertion of Advanced Locking Screws (T2 Alpha Femur Retrograde Intramedullary Nailing System): Report of Two Cases and Identifying Causes and Prevention

**DOI:** 10.3390/jcm13082393

**Published:** 2024-04-19

**Authors:** Takashi Higuchi, Atsushi Taninaka, Rikuto Yoshimizu, Katsuhiro Hayashi, Shinji Miwa, Norio Yamamoto, Hiroyuki Tsuchiya, Satoru Demura

**Affiliations:** 1 Department of Orthopaedic Surgery, Kanazawa Red Cross Hospital, 2-251, Minma, Kanazawa 921-8162, Ishikawa, Japan; atsushi880628@yahoo.co.jp; 2 Department of Orthopaedic Surgery, Graduate School of Medical Sciences, Kanazawa University, 13-1, Takaramachi, Kanazawa 921-8641, Ishikawa, Japan; cogito_ergo_sum_1010@icloud.com (R.Y.); khayashi830@gmail.com (K.H.); miwapoti@yahoo.co.jp (S.M.); norinori-ya@wing.ocn.ne.jp (N.Y.); tsuchiya62958@gmail.com (H.T.); msdemura@gmail.com (S.D.)

**Keywords:** osteoporosis, elderly patient, intraoperative fracture, retrograde intramedullary nail, supracondylar femoral fractures, advanced locking screws, fragility fractures

## Abstract

**Background**: Recently, the T2 alpha nailing system (Stryker, Inc.), which has advanced locking screws that can attach a screw to a rod, has been used worldwide and is expected to improve fracture fixation. We analyzed two cases of supracondylar femoral fractures in older adult patients, in which intraoperative fractures occurred during the insertion of advanced locking screws of the T2 alpha femur retrograde intramedullary nail. **Case presentation**: A 93-year-old and an 82-year-old woman each underwent T2 alpha femur retrograde nail fixation for supracondylar femur fractures at separate hospitals, and advanced locking screws were used as the proximal transverse locking screws. In both patients, a fracture line was observed at the proximal screw postoperatively, and the fractures were refixed with distal cable wiring and/or femoral distal plates. The patients were subsequently discharged from the same facility with no remarkable pain. **Conclusions**: When inserting advanced locking screws, it is necessary to enlarge the screw hole in the near-bone cortex with a counterbore drill, which might add torque to the bone cortex that could result in fractures. If the sleeve is distant from the bone, the counterbore drill will not reach the bone, the screw hole will not expand, and the insertion of advanced locking screws will apply a strong torque to the bone cortex and may result in fracture. Moreover, it is important to confirm that the counterbore drill is securely inserted under fluoroscopy and to carefully enlarge the bony foramen manually to prevent fractures during screw insertion.

## 1. Introduction

As more people live longer, the number of fragility fractures is inevitably increasing. The treatment of fragility fractures is sometimes challenging for orthopedic surgeons. This is because, in addition to the underlying bone fragility, the presence of frailty, dementia, and other medical complications can lead to perioperative and postoperative complications that require a high degree of vigilance. Intramedullary nails (IMNs) are often preferred for older adults for the following reasons: small skin incision and minimally invasive, less impaired blood flow, and periosteal damage at the fracture site, which is advantageous for bone healing; spontaneous correction of alignment when inserting nails; and mechanical stability due to the presence of the implant in the medullary cavity center [1,2]. However, the use of IMNs for osteoporotic bone repair has the following potential risks: the bone cortex is quite thin, and the medullary cavity is wide, especially in the metaphyseal region in older adults. Thus, in addition to poor alignment or poor reduction, the “windshield-wiper” effect, which results in the formation of an osteolytic lesion around the implant tip as a result of the implant oscillating in the bone while the implant fixation is unstable, becomes problematic [3,4].

The T2 alpha nailing system (Stryker Corp., Kalamazoo, MI, USA), developed to increase construct stability in unstable fracture patterns and poor bone quality, is now widely used. According to the manufacturer, the advanced locking screws in this system are designed to limit the relative axial and angular movement of the nail and screw, which is achieved by their threaded interface. The advanced locking screws prevent screw backout. While this system is advantageous for the initial fixation of fractures in older adults, improper use results in high insertion torques, which can lead to fractures in fragile bones. This report demonstrates two cases of supracondylar femoral fractures in older adult patients, in which intraoperative fractures occurred during the insertion of advanced locking screws of the T2 alpha femur retrograde intramedullary nails. Our discussion offers possible causes of intraoperative fractures and highlights steps to prevent them from occurring when using this system.

## 2. Case Presentation

### 2.1. Patient 1

**Figure 1:**
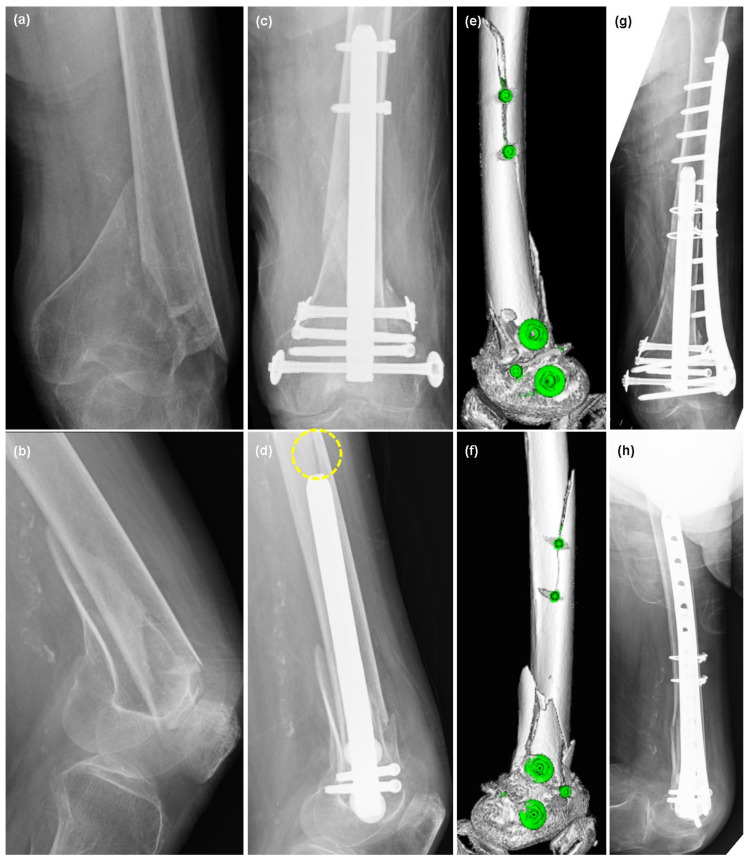
A 93-year-old female with a left supracondylar femoral fracture. (**a**) Preoperative frontal X-ray image of the distal femur. (**b**) Lateral image. (**c**) Postoperative X-ray frontal image. (**d**) Lateral image. The dotted circle shows the fracture line. (**e**,**f**) Postoperative CT images. The fracture was along the proximal advanced locking screws. (**g**) Re-fixation with distal femoral lateral plate (LCP DF, DePuy Synthes) and cable wires (DePuy Synthes). Front image. (**h**) Lateral image.

A 93-year-old female, who was institutionalized at the level of full daily living assistance due to her dementia, heart failure, severe hip deformity, and hypothyroidism, injured her left lower leg while changing a diaper. The patient was rushed to our hospital and diagnosed with a left femoral supracondylar fracture (Arbeitsgemeinschaft für Osteosynthesefragen [AO] fracture classification: 33-A2, Figure 1a,b). Considering the patient’s background and her bone quality, minimally open reduction and intramedullary nail fixation using T2 alpha femur retrograde intramedullary nails (Stryker Corp., Kalamazoo, MI, USA) were performed (Figure 1c,d). Small skin incisions were made to repair the fracture with forceps; a longitudinal incision was made in the middle of the patellar tendon; and an intramedullary nail was inserted anterior to Blumensaat’s line. The distal side of the fracture was fixed bicortically with two condylar screws and two advanced locking screws (Figure 1c,d). The diaphyseal (proximal) side was fixed using two advanced locking screws (Figure 1c,d). Postoperative radiographs revealed a fracture line originating from the proximal screws (Figure 1d), and computed tomography (CT) revealed a fracture with dislocation around the screws (Figure 1e,f). One week postoperatively, the patient underwent revision surgery with a distal femur lateral plate (LCP DF, DePuy Synthes, Inc., Raynham, MA, USA) and two cable wires (Depuy Synthes) (Figure 1g,h). The patient then achieved the same level of daily living as before the injury and was discharged to the same long-term care facility.

### 2.2. Patient 2

**Figure 2:**
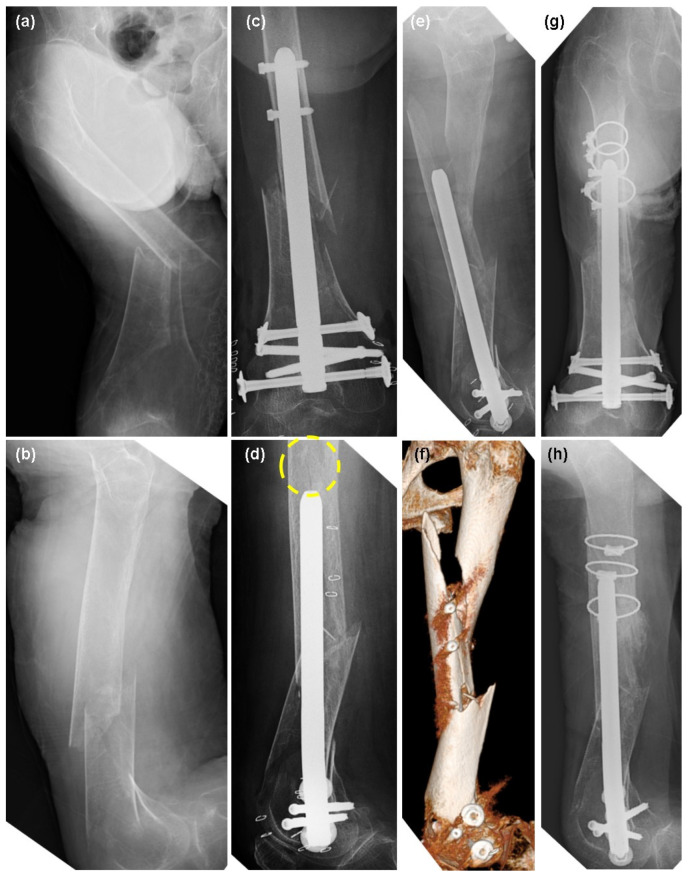
An 82-year-old female with a right supracondylar femoral fracture. (**a**) Preoperative frontal X-ray image of the distal femur. (**b**) Lateral image. (**c**) Postoperative frontal X-ray image. (**d**) Lateral image. The dotted circle shows the fracture line. (**e**) X-ray lateral image at one week postoperatively. A dislocated fracture was observed. (**f**) Postoperative CT image at one week postoperatively. The fracture was along the proximal advanced locking screws. (**g**) Re-fixation with cable wires (DePuy Synthes). Front image. (**h**) Lateral image.

An 82-year-old female with severe dementia and osteoporosis, who had no relatives and was staying in a facility, twisted her right lower leg while changing a diaper and was taken to another hospital. The patient was diagnosed with a right femoral supracondylar fracture (33-A3, Figure 2a,b), which was fixed with minimal open reduction and intramedullary fixation using T2 alpha femur retrograde nails (Stryker Corp., Figure 2c,d). The distal side of the fracture was fixed bicortically with two condylar screws and two conventional locking screws (Figure 2c,d). The proximal side was fixed using two advanced locking screws (Figure 2c,d). One-week postoperative radiographic imaging revealed a fracture proximal to the intramedullary nail (Figure 2e), and a CT scan revealed a fracture near the advanced locking screws (Figure 2f). A review of the immediate postoperative radiographs showed fracture lines originating from advanced locking screws, similar to Patient 1 (Figure 1d). Pending improvement in her general condition, the patient underwent revisionary surgery with two cable wires (Depuy Synthes) three weeks after the initial surgery (Figure 2g,h). The patient was subsequently discharged to the same residential facility with no remarkable pain.

## 3. Discussion

Supracondylar femoral fractures in older adults are often based on osteoporosis and are caused by low-energy trauma such as indoor falls [5,6,7,8,9]. This fracture type is a periarticular fracture, which requires range-of-motion training based on accurate reduction and firm fixation, whereas older adults have poor bone quality and instability involving the fracture site, which often makes internal fixation difficult [6,10]. It has been reported that early surgery within two days of hospital admission for distal femur fracture in older adult patients reduces postoperative complications including acute coronary syndrome, and total hospitalization costs [10]. Plates, screws, or IMNs are often the fixation materials of choice [5,8,9,11]. 

Plate fixations have an advantage in terms of anatomical reduction and in that it does not damage the joint cartilage or patellar tendon; however, their reliance on screw fixtures within the cancellous bone, which in older adult patients has less trabecular bone, leading to lower strength in fixation, and the deployment of the vastus lateralis muscle can cause knee contractures [5,8,9]. Plate fixations are usually more invasive, and the postoperative infection rate is reported to be significantly higher than that of intramedullary nail fixation in a systematic review and meta-analysis of 936 patients with distal femur fractures in 16 studies [8]. The minimally invasive plate osteosynthesis (MIPO) technique can overcome these problems because it requires less skin and soft-tissue dissection and preserves the fracture hematoma and periosteal blood supply, resulting in undisturbed callus bone healing [12,13]. However, closed reduction in MIPO is technically demanding and may rather lead to malreduction or malalignment [12,13,14]. 

IMN fixation can be performed with a small dermal incision, and a tourniquet can be used in many cases of supracondylar femoral fractures, which reduces blood loss [5,8,9]. It has been reported that closed reduction with IMN results in significantly less sterile inflammation, which is triggered in correspondence with the degree of tissue damage sustained after a surgical procedure and can lead to the major postoperative complications in the elderly patients [15]. Moreover, the use of a condylar screw allows for cortical bone-to-cortical bone fixation and has an advantage in initial fixation strength due to the mechanical stability provided by the presence of the implant in the center of the medullary cavity in IMN fixation [16]. IMN fixation is reported to have significantly lower non-union rates in a systematic review [8]. In contrast, knee contracture and the development of osteoarthritis are problematic because IMN fixation requires incision of the patellar tendon and joint capsule [7]. The postoperative range of motion of the knee joint is reported to be higher in plate fixation in the systematic review [8]. In addition, IMN use is not indicated for intra-articular fractures with dislocation (AO:C3) or coronal fractures of the femoral condyle (Hoffa fracture, AO:B3). However, these points may not be an issue in supracondylar femoral fractures in older adults because most injuries are caused by low-energy trauma resulting in A-type fractures in terms of AO classifications, and older adult patients often already have osteoarthritis of the knee pre-trauma. A remaining issue for IMN use in supracondylar femoral fractures in older adults has reduced fixation due to instability of the IMNs in bone, as older adults typically have a thin and fragile bone cortex with a wide medullary cavity, and the T2 alpha nailing system is expected to be a solution to this issue.

**Figure 3:**
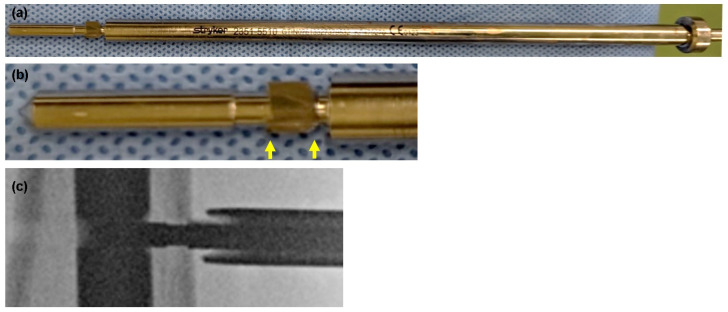
(**a**) Counterbore drill (electric, Striker). The shank on the hand side of the drill is thicker to interfere with the drill sleeve. (**b**) Enlarged photo of the drill tip. The blade part is short (arrows). Intraoperative fluoroscopic image. The blade part is not inside the near cortex, although most of the drill tip is inside the nail and the bone. (**c**) The blade of the counterbore drill, which is difficult to identify under the fluoroscopy, does not reach the bone because the sleeve is distant from the bone.

**Figure 4:**
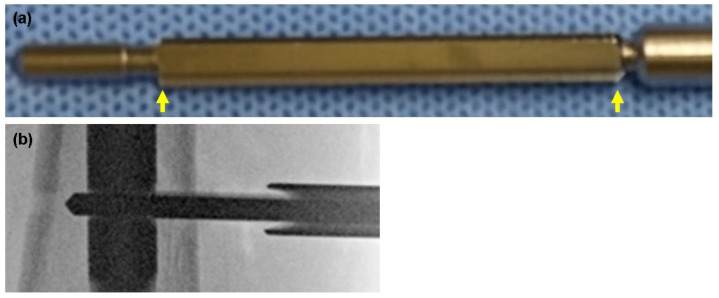
(**a**) Drill tip of a manual counterbore drill. The blade part is longer than that of the electric counterbore drill (arrows). (**b**) The blade of the drill is absolutely inside the near cortex and enlarges the drill hole; nevertheless, the drill sleeve is apart from the bone.

The advanced locking screws are designed with oversized threads that engage with the internal threads of the T2 Alpha nails while maintaining bicortical purchase to add axial and angular stability. Therefore, when inserting the advanced locking screws into the bone and the T2 alpha nails, the screw hole of the near-cortex should be enlarged using the dedicated counterbore drill after drilling both cortices with a normal drill bit and determining screw lengths. In this process, an electric counterbore drill (Figure 3) was used in the two cases, and one possibility is that the torque of the drill caused the fracture because the patients had severe osteoporosis and the cortical bones were quite thin and fragile in both patients. However, there is no data on how much torque should be applied in this process. The use of a manual counterbore drill (Figure 4) is an option, which may be suitable for adjusting the torque applied to such osteoporotic bones, although there is no data to support this either.

Another possible cause of intraoperative fractures is failure to expand the cortex with a counterbore drill. The advanced locking screw insertion torque, when properly over-drilled, was the same as or less than the normal full-thread locking screw insertion torque (data not published). If this over-drilling process is not carried out appropriately, significant torque will be applied to the near-cortex when the advanced locking screw is inserted, which could lead to fractures. The counterbore drill used in these cases had a blade-less component in front of the blade for centering within the pre-drilled hole and the nail hole (Figure 3a,b). The blade length of the counterbore drill is designed to be short to avoid over-drilling of cancellous bone other than the near-cortex as much as possible (Figure 3b). The design of the drill makes it difficult to observe if the blade is in the cortex, even when using fluoroscopy (Figure 3c). In addition, the shank on the hand side of the drill is thicker in diameter to interfere with the drill sleeve and prevent the drill from penetrating cancellous bone unnecessarily (Figure 3a). If the sleeve is distant from the bone, the blade of the counterbore drill cannot reach the bone (Figure 3c). Using an electric drill makes it more difficult to check whether the blade is in the cortex because there is little sensation of cutting. The above may have caused the counterbore drill to fail to cut the cortical bone, and when the advanced locking screw was inserted, significant torque was applied, leading to an intraoperative fracture. To prevent this, a manual counterbore drill may be preferable because the blade part of the drill is much longer (Figure 4a) and can, therefore, reach the cortex sufficiently and is easy to confirm with fluoroscopy (Figure 4b). This drill is even more reliable because it is manually operated, and the surgeon can feel sensations of the bone being cut. Finally, the torque applied during screw insertion should be carefully monitored, and preparation should be made to switch to a standard locking screw or to drill the near-cortex again with a manual counterbore drill if excessive force is required. The T2 alpha series is a new intramedullary nail system and other similar cases should be searched to clarify the causal relationship with intraoperative fractures.

## 4. Conclusions

T2 alpha femur retrograde nails were used for two separate supracondylar femoral fractures in two older adult patients who experienced intraoperative fractures originating from advanced locking screws and following re-fixations. The advanced locking screw of the T2 alpha series is expected to improve fracture fixation in older adult patients with a large medullary cavity by engaging with the screw hole of the nail to stabilize the nail and screw. When inserting the advanced locking screw, the drill hole in the near-cortex should be carefully expanded to avoid fractures, observing if the blade of the counterbore drill is in the cortex or if the sleeve is distant from the cortex with fluoroscopy, and monitoring the screw insertion torque, with preparations to switch to a standard locking screw if excessive force is required. The manual counterbore drill may be safer and more reliable for less-experienced surgeons when using this system to gain rigid fixation in older adult patients and not to generate intraoperative fractures.

## Data Availability

The data presented in this study are available on request from the corresponding author. The data are not publicly available due to privacy.
